# Supplementary material for: Differences in self-perception of productivity and mental health among the STEMM-field scientists during the COVID-19 pandemic by sex and status as a parent: A survey in six languages
Source: PLoS One. 2022 Jul 1;17(7):e0269834. doi: 10.1371/journal.pone.0269834 (PMC9249185; doi:10.1371/journal.pone.0269834)
Supplement: S1 Table — (DOCX) [file pone.0269834.s001.docx]

**S1 Table. List of countries where the survey participants reside.**

| Name | Participants | Name | Participants | Name | Participants |
| --- | --- | --- | --- | --- | --- |
| Albania | 7 | Honduras | 2 | Poland | 56 |
| Algeria | 5 | Hong Kong (S.A.R.) | 28 | Portugal | 49 |
| Angola | 1 | Hungary | 19 | Qatar | 6 |
| Argentina | 25 | Iceland | 4 | Romania | 33 |
| Australia | 139 | India | 79 | Russian Federation | 53 |
| Austria | 33 | Indonesia | 22 | Rwanda | 1 |
| Azerbaijan | 2 | Iran | 17 | Saint Kitts and Nevis | 1 |
| Baharain | 3 | Iraq | 9 | Saint Lucia | 1 |
| Bangladesh | 9 | Ireland | 8 | Saudi Arabia | 16 |
| Belarus | 3 | Israel | 11 | Senegal | 1 |
| Belgium | 37 | Italy | 138 | Serbia | 10 |
| Benin | 1 | Jamaica | 3 | Sierra Leone | 1 |
| Bhutan | 1 | Japan | 63 | Singapore | 17 |
| Bosnia and Herzegovina | 9 | Jordan | 6 | Slovakia | 12 |
| Brazil | 127 | Kazakhstan | 4 | Slovenia | 9 |
| Brunei Darussalam | 6 | Kenya | 9 | South Africa | 34 |
| Bulgaria | 9 | Kuwait | 3 | South Korea | 181 |
| Burkina Faso | 2 | Kyrgyzstan | 1 | Spain | 140 |
| Cambodia | 2 | Lao People's Democratic Republic | 1 | Sri Lanka | 5 |
| Cameroon | 5 | Latvia | 6 | Sudan | 1 |
| Canada | 153 | Lebanon | 4 | Sweden | 68 |
| Chana | 3 | Libyan Arab Jamahiriya | 1 | Switzerland | 60 |
| Chile | 28 | Lithuania | 7 | Tajikistan | 1 |
| China | 344 | Luxembourg | 5 | Thailand | 35 |
| Colombia | 8 | Madagascar | 1 | The former Yugoslav Republic of Macedonia | 3 |
| Congo, Republic of the | 2 | Malawi | 5 | Togo | 2 |
| Costa Rica | 5 | Malaysia | 21 | Trinidad and Tobago | 1 |
| Côte d'Ivoire | 5 | Mauritius | 1 | Tunisia | 7 |
| Croatia | 29 | Mexico | 62 | Turkey | 71 |
| Cyprus | 1 | Monaco | 1 | Uganda | 5 |
| Czech Republic | 27 | Mongolia | 2 | Ukraine | 17 |
| Democratic Republic of the Congo | 1 | Montenegro | 1 | United Arab Emirates | 1 |
| Denmark | 15 | Morocco | 9 | United Kingdom of Great Britain and Northern Ireland | 151 |
| Ecuador | 9 | Mozambique | 1 | United Republic of Tanzania | 2 |
| Egypt | 15 | Namibia | 1 | United States of America | 1171 |
| El Salvador | 1 | Nepal | 12 | Uruguay | 4 |
| Estonia | 7 | Netherlands | 50 | Uzbekistan | 6 |
| Ethiopia | 12 | New Zealand | 35 | Venezuela, Bolivarian Republic of... | 3 |
| Finland | 29 | Nigeria | 22 | Viet Nam | 5 |
| France | 101 | Norway | 25 | Yemen | 1 |
| Gabon | 2 | Oman | 1 | Zambia | 1 |
| Georgia | 1 | Pakistan | 19 | Zimbabwe | 2 |
| Germany | 235 | Panama | 2 | Other | 11 |
| Greece | 36 | Peru | 11 |  | |
| Guinea | 1 | Philippines | 13 |  |  |
