# Supplementary material for: Differences in self-perception of productivity and mental health among the STEMM-field scientists during the COVID-19 pandemic by sex and status as a parent: A survey in six languages
Source: PLoS One. 2022 Jul 1;17(7):e0269834. doi: 10.1371/journal.pone.0269834 (PMC9249185; doi:10.1371/journal.pone.0269834)
Supplement: S2 Table — (DOCX) [file pone.0269834.s002.docx]

**S2 Table. Results of multivariate regression analysis for DASS-21 scores of depression, anxiety, and stress (*n*=3,893).**

| Variable | Beta (95% CI) | | |
| --- | --- | --- | --- |
|  | DASS – Depression score | DASS – anxiety score | DASS – Stress score |
| Employment |  |  |  |
| Currently unemployed | Reference | Reference | Reference |
| Currently employed | 0.24 (-1.27, 1.76) | -0.12 (-1.16, 0.92) | 0.23 (-1.23, 1.70) |
| Marital status |  |  |  |
| Single | Reference | Reference | Reference |
| Divorced/widowed/separated | -1.01 (-2.05, 0.03)† | -0.78 (-1.49, -0.06)* | -1.23 (-2.23, -0.22)* |
| Living with a partner | -0.92 (-1.81, -0.03)* | -0.32 (-0.93, 0.29) | 0.54 (-0.32, 1.40) |
| Married | -1.39 (-2.09, -0.68)* | -0.10 (-0.59, 0.38) | -0.24 (-0.93, 0.44) |
| Early-career status |  |  |  |
| No | Reference | Reference | Reference |
| Yes | 0.30 (-0.25, 0.85) | 0.09 (-0.29, 0.47) | 0.13 (-0.40, 0.66) |
| Working in the fileds involving lab experiments, bench science work, wet-science, and living organisms |  |  |  |
| No | Reference | Reference | Reference |
| Yes | 0.29 (-0.16, 0.74) | 0.33 (0.02, 0.64)* | 0.03 (-0.4, 0.47) |
| Sex |  |  |  |
| Male | Reference | Reference | Reference |
| Female | 0.21 (-0.25, 0.67) | 0.01 (-0.31, 0.33) | 0.90 (0.45, 1.35)* |
| Status as a parent of children age <18 years |  |  |  |
| No | Reference | Reference | Reference |
| Yes | -0.36 (-0.95, 0.23) | -0.14 (-0.54, 0.26) | 0.10 (-0.47, 0.67) |
| Age (years) |  |  |  |
| 19–29 | Reference | Reference | Reference |
| 30–59 | -1.39 (-2.61, -0.18)* | -1.72 (-2.55, -0.88)* | -0.36 (-1.53, 0.82) |
| ≥60 | -2.99 (-4.35, -1.64)* | -2.64 (-3.57, -1.71)* | -1.75 (-3.06, -0.44)* |
| Loss of family due to COVID-19 |  |  |  |
| Yes | Reference | Reference | Reference |
| No | -0.40 (-0.9, 0.11) | -0.60 (-0.95, -0.26)* | -0.44 (-0.92, 0.05)† |
| Prefer not to say | 3.49 (0.75, 6.23)* | 1.53 (-0.35, 3.41) | 1.54 (-1.11, 4.19) |
| Diagnosis of mental health problems in last 12 months |  |  |  |
| No | Reference | Reference | Reference |
| Yes | 5.52 (4.92, 6.11)* | 3.86 (3.45, 4.27)* | 4.92 (4.35, 5.49)* |
| Working with COVID-19 confirmed patients or in place with high contact with COVID-19 patients |  |  |  |
| Yes | Reference | Reference | Reference |
| No | -0.32 (-1.01, 0.36) | -0.38 (-0.85, 0.09) | -0.57 (-1.23, 0.1)† |
| Prefer not to say | 0.56 (-1.61, 2.73) | 1.36 (-0.14, 2.85)† | -0.38 (-2.48, 1.72) |
| Changes in the number of work hours |  |  |  |
| Significantly decreased | Reference | Reference | Reference |
| Slightly decreased | -1.55 (-2.57, -0.53)* | -1.38 (-2.08, -0.68)* | -2.03 (-3.02, -1.05)* |
| No change | -2.24 (-3.20, -1.28)* | -1.17 (-1.83, -0.51)* | -2.23 (-3.16, -1.3)* |
| Slightly increased | -1.92 (-2.88, -0.97)* | -0.97 (-1.63, -0.31)* | -1.29 (-2.22, -0.37)* |
| Significantly increased | -1.45 (-2.45, -0.45)* | -0.44 (-1.12, 0.25) | -0.06 (-1.03, 0.90) |
| Losing job |  |  |  |
| No | Reference | Reference | Reference |
| Yes | -0.54 (-2.68, 1.61) | -1.99 (-3.46, -0.52)* | -1.09 (-3.17, 0.98) |
| Loss of job of spouse/partner |  |  |  |
| No | Reference | Reference | Reference |
| Yes | 0.63 (-0.56, 1.81) | 1.45 (0.64, 2.27)* | 0.54 (-0.60, 1.69) |
| Experiencing salary cut or paycheck delay |  |  |  |
| No | Reference | Reference | Reference |
| Yes | 0.26 (-0.46, 0.98) | 0.37 (-0.13, 0.86) | -0.19 (-0.89, 0.50) |
| Experiencing financial difficulties |  |  |  |
| No | Reference | Reference | Reference |
| Yes | 0.87 (0.07, 1.67)* | 1.44 (0.9, 1.99)* | 0.53 (-0.24, 1.30) |
| Experiencing reduced contract renewal or other changes in job security |  |  |  |
| No | Reference | Reference | Reference |
| Yes | 1.2 (0.41, 1.99)* | 0.78 (0.24, 1.32)* | 1.65 (0.89, 2.41)* |
| Considering early retirement or being forced to retire |  |  |  |
| No | Reference | Reference | Reference |
| Yes | 2.86 (1.8, 3.92)* | 0.99 (0.27, 1.72)* | 1.37 (0.35, 2.40)* |
| Restricted access to campus, office, labs, field work, or other facilities |  |  |  |
| No | Reference | Reference | Reference |
| Yes | 0.39 (-0.23, 1.01) | -0.19 (-0.61, 0.23) | 0.59 (0.00, 1.19)† |
| Decreased or delayed funding for research |  |  |  |
| No | Reference | Reference | Reference |
| Yes | 0.47 (-0.04, 0.97)† | 0.61 (0.26, 0.96)* | 0.44 (-0.05, 0.93)† |
| Delayed research work |  |  |  |
| No | Reference | Reference | Reference |
| Yes | 0.22 (-0.26, 0.69) | -0.17 (-0.5, 0.15) | 0.08 (-0.37, 0.54) |
| Challenge in recruitment of research participants |  |  |  |
| No | Reference | Reference | Reference |
| Yes | -0.26 (-0.73, 0.21) | -0.21 (-0.53, 0.11) | 0.35 (-0.10, 0.80) |
| Elimination or restructuring of department of institution |  |  |  |
| No | Reference | Reference | Reference |
| Yes | 0.56 (-0.14, 1.25) | 0.70 (0.22, 1.18)* | 0.47 (-0.21, 1.14) |
| Poor workspace or work condition at home |  |  |  |
| No | Reference | Reference | Reference |
| Yes | 2.01 (1.51, 2.50)* | 0.60 (0.26, 0.94)* | 2.06 (1.58, 2.54)* |
| Restriction on work travels |  |  |  |
| No | Reference | Reference | Reference |
| Yes | -0.47 (-1.03, 0.09) | -0.49 (-0.87, -0.10)* | -0.31 (-0.85, 0.23) |
| Increased demands for childcare/eldercare |  |  |  |
| No | Reference | Reference | Reference |
| Yes | -0.25 (-0.84, 0.34) | -0.19 (-0.59, 0.22) | 0.44 (-0.13, 1.01) |
| Increased demands for domestic work |  |  |  |
| No | Reference | Reference | Reference |
| Yes | 0.33 (-0.15, 0.81) | 0.48 (0.15, 0.80)* | 0.58 (0.12, 1.04)* |

*: Significant at a significance level of 0.05. †: Significant at a significance level of 0.1. Participants with missing data were omitted.
