# Supplementary material for: Differences in self-perception of productivity and mental health among the STEMM-field scientists during the COVID-19 pandemic by sex and status as a parent: A survey in six languages
Source: PLoS One. 2022 Jul 1;17(7):e0269834. doi: 10.1371/journal.pone.0269834 (PMC9249185; doi:10.1371/journal.pone.0269834)
Supplement: S3 Table — (DOCX) [file pone.0269834.s003.docx]

**S3 Table. Results of multivariate regression analysis for DASS-21 scores of depression, anxiety, and stress for the participants in Africa (n=162).**

| Variable | Beta (95% CI) | | |
| --- | --- | --- | --- |
|  | DASS – Depression score | DASS – anxiety score | DASS – Stress score |
| Employment |  |  |  |
| Currently unemployed | Reference | Reference | Reference |
| Currently employed | -2.18 (-12.08, 7.71) | -2.54 (-10.64, 5.57) | -0.85 (-12.11, 10.41) |
| Marital status |  |  |  |
| Single | Reference | Reference | Reference |
| Divorced/widowed/separated | -7.76 (-15.22, -0.30)* | -4.15 (-10.24, 1.94) | -4.58 (-11.91, 2.75) |
| Living with a partner | 2.85 (-4.43, 10.13) | 3.02 (-2.5, 8.53) | 6.51 (-0.13, 13.15)† |
| Married | -2.26 (-7.52, 2.99) | -0.83 (-4.96, 3.3) | 0.65 (-4.26, 5.56) |
| Early-career status |  |  |  |
| No | Reference | Reference | Reference |
| Yes | 2.03 (-2.27, 6.33) | 0.02 (-3.35, 3.39) | -0.06 (-4.24, 4.12) |
| Working in the fields involving lab experiments, bench science work, wet-science, and living organisms |  |  |  |
| No | Reference | Reference | Reference |
| Yes | 2.40 (-0.86, 5.66) | 1.07 (-1.41, 3.55) | -0.15 (-3.23, 2.92) |
| Sex |  |  |  |
| Male | Reference | Reference | Reference |
| Female | 1.70 (-1.94, 5.35) | 0.91 (-1.85, 3.67) | 2.02 (-1.35, 5.39) |
| Status as a parent of children age <18 years |  |  |  |
| No | Reference | Reference | Reference |
| Yes | 0.37 (-4.25, 4.98) | 1.66 (-2.01, 5.34) | 1.07 (-3.32, 5.45) |
| Age (years) |  |  |  |
| 19–29 | Reference | Reference | Reference |
| 30–59 | -2.12 (-8.66, 4.42) | -9.37 (-14.71, -4.04)* | -6.94 (-13.29, -0.60)* |
| ≥60 | -4.08 (-13.02, 4.85) | -8.60 (-15.86, -1.33)* | -6.42 (-15.5, 2.65) |
| Loss of family due to COVID-19 |  |  |  |
| Yes | Reference | Reference | Reference |
| No | -3.48 (-6.68, -0.29)* | -2.39 (-5.00, 0.22)† | -3.69 (-6.81, -0.57)* |
| Prefer not to say | -6.82 (-23.78, 10.15) | -0.49 (-14.37, 13.39) | 6.47 (3.03, 9.92)* |
| Diagnosis of mental health problems in last 12 months |  |  |  |
| No | Reference | Reference | Reference |
| Yes | 9.96 (6.38, 13.54)* | 7.24 (4.38, 10.1)* | 0.10 (-3.43, 3.62) |
| Working with COVID-19 confirmed patients or in place with high contact with COVID-19 patients |  |  |  |
| Yes | Reference | Reference | Reference |
| No | -0.37 (-4.06, 3.32) | -0.51 (-3.43, 2.4) | -5.83 (-15.41, 3.74) |
| Prefer not to say | -2.34 (-12.17, 7.48) | -9.60 (-19.35, 0.16)† | 0.35 (-6.53, 7.24) |
| Changes in the number of work hours |  |  |  |
| Significantly decreased | Reference | Reference | Reference |
| Slightly decreased | -0.5 (-7.59, 6.58) | 0.99 (-4.77, 6.76) | -0.42 (-7.05, 6.21) |
| No change | -1.79 (-8.59, 5.01) | -2.51 (-8.15, 3.14) | -0.40 (-7.04, 6.25) |
| Slightly increased | -1.85 (-8.68, 4.99) | 0.22 (-5.31, 5.75) | 6.28 (-0.16, 12.72)† |
| Significantly increased | 4.15 (-2.67, 10.97) | 2.64 (-2.76, 8.04) | 1.69 (-6.23, 9.61) |
| Losing job |  |  |  |
| No | Reference | Reference | Reference |
| Yes | 5.78 (-2.00, 13.57) | 8.61 (2.00, 15.23)* | 0.58 (-6.60, 7.75) |
| Loss of job of spouse/partner |  |  |  |
| No | Reference | Reference | Reference |
| Yes | 2.52 (-4.87, 9.91) | 3.60 (-2.38, 9.58) | -0.82 (-4.74, 3.09) |
| Experiencing salary cut or paycheck delay |  |  |  |
| No | Reference | Reference | Reference |
| Yes | -0.09 (-4.07, 3.89) | -1.14 (-4.41, 2.12) | 2.31 (-1.16, 5.78) |
| Experiencing financial difficulties |  |  |  |
| No | Reference | Reference | Reference |
| Yes | 0.67 (-2.87, 4.22) | 1.34 (-1.60, 4.27) | -1.26 (-5.24, 2.73) |
| Experiencing reduced contract renewal or other changes in job security |  |  |  |
| No | Reference | Reference | Reference |
| Yes | 0.99 (-3.05, 5.02) | -1.58 (-4.82, 1.67) | -0.60 (-6.75, 5.54) |
| Considering early retirement or being forced to retire |  |  |  |
| No | Reference | Reference | Reference |
| Yes | 6.60 (-0.06, 13.26)† | 2.17 (-2.93, 7.28) | 3.76 (-0.14, 7.66)† |
| Restricted access to campus, office, labs, field work, or other facilities |  |  |  |
| No | Reference | Reference | Reference |
| Yes | -0.64 (-4.61, 3.33) | 0.25 (-3.02, 3.51) | -0.30 (-3.44, 2.85) |
| Decreased or delayed funding for research |  |  |  |
| No | Reference | Reference | Reference |
| Yes | -0.47 (-3.72, 2.78) | 1.47 (-1.21, 4.16) | -3.57 (-7.23, 0.10) † |
| Delayed research work |  |  |  |
| No | Reference | Reference | Reference |
| Yes | -0.21 (-4.02, 3.60) | -3.30 (-6.35, -0.25)* | -1.51 (-5.00, 1.98) |
| Challenge in recruitment of research participants |  |  |  |
| No | Reference | Reference | Reference |
| Yes | -2.08 (-5.67, 1.50) | -0.21 (-3.09, 2.67) | 2.50 (-1.63, 6.64) |
| Elimination or restructuring of department of institution |  |  |  |
| No | Reference | Reference | Reference |
| Yes | -0.40 (-4.79, 3.99) | 2.76 (-0.76, 6.27) | 0.99 (-2.29, 4.27) |
| Poor workspace or work condition at home |  |  |  |
| No | Reference | Reference | Reference |
| Yes | 0.51 (-2.96, 3.97) | 0.42 (-2.30, 3.15) | 1.34 (-2.80, 5.48) |
| Restriction on work travels |  |  |  |
| No | Reference | Reference | Reference |
| Yes | 0.16 (-4.03, 4.36) | -0.20 (-3.65, 3.24) | 3.46 (-0.18, 7.10) |
| Increased demands for childcare/eldercare |  |  |  |
| No | Reference | Reference | Reference |
| Yes | 3.29 (-0.41, 7.00)† | 2.71 (-0.31, 5.72)† | -0.35 (-3.75, 3.04)† |
| Increased demands for domestic work |  |  |  |
| No | Reference | Reference | Reference |
| Yes | -1.90 (-5.34, 1.55) | -0.26 (-3.04, 2.52) | -0.26 (-3.04, 2.52) |

*: Significant at a significance level of 0.05. †: Significant at a significance level of 0.1. Participants with missing data were omitted.
