# Supplementary material for: Differences in self-perception of productivity and mental health among the STEMM-field scientists during the COVID-19 pandemic by sex and status as a parent: A survey in six languages
Source: PLoS One. 2022 Jul 1;17(7):e0269834. doi: 10.1371/journal.pone.0269834 (PMC9249185; doi:10.1371/journal.pone.0269834)
Supplement: S4 Table — (DOCX) [file pone.0269834.s004.docx]

**S4 Table. Results of multivariate regression analysis for DASS-21 scores of depression, anxiety, and stress for the participants in Asia (*n*=951).**

| Variable | Beta (95% CI) | | |
| --- | --- | --- | --- |
|  | DASS – Depression score | DASS – anxiety score | DASS – Stress score |
| Employment |  |  |  |
| Currently unemployed | Reference | Reference | Reference |
| Currently employed | -1.84 (-7.33, 3.65) | -2.29 (-6.44, 1.86) | -1.36 (-7.08, 4.35) |
| Marital status |  |  |  |
| Single | Reference | Reference | Reference |
| Divorced/widowed/separated | -4.00 (-6.92, -1.07)* | -2.31 (-4.56, -0.06)* | -3.01 (-6.06, 0.04)† |
| Living with a partner | -2.22 (-5.79, 1.35) | -1.62 (-4.32, 1.09) | -2.07 (-5.87, 1.73) |
| Married | -2.68 (-4.36, -1.00)* | -0.77 (-2.04, 0.50) | -0.80 (-2.55, 0.95) |
| Early-career status |  |  |  |
| No | Reference | Reference | Reference |
| Yes | 0.85 (-0.49, 2.19) | 0.73 (-0.28, 1.75) | 0.87 (-0.52, 2.26) |
| Working in the fields involving lab experiments, bench science work, wet-science, and living organisms |  |  |  |
| No | Reference | Reference | Reference |
| Yes | 0.58 (-0.43, 1.60) | 0.08 (-0.69, 0.85) | -0.16 (-1.22, 0.90) |
| Sex |  |  |  |
| Male | Reference | Reference | Reference |
| Female | -0.45 (-1.60, 0.70) | -0.15 (-1.02, 0.72) | -0.57 (-1.77, 0.63) |
| Status as a parent of children age <18 years |  |  |  |
| No | Reference | Reference | Reference |
| Yes | 0.24 (-1.01, 1.50) | 0.31 (-0.65, 1.26) | 0.60 (-0.71, 1.92) |
| Age (years) |  |  |  |
| 19–29 | Reference | Reference | Reference |
| 30–59 | -1.62 (-4.34, 1.10) | -2.06 (-4.12, 0.00)† | -1.57 (-4.45, 1.31) |
| ≥60 | -3.20 (-6.43, 0.03)† | -1.92 (-4.35, 0.52) | -3.10 (-6.52, 0.31)† |
| Loss of family due to COVID-19 |  |  |  |
| Yes | Reference | Reference | Reference |
| No | 0.66 (-0.80, 2.12) | 0.10 (-1.01, 1.20) | 0.24 (-1.27, 1.76) |
| Prefer not to say | 8.87 (3.83, 13.90)* | 3.69 (-0.14, 7.51)† | 5.15 (-0.09, 10.39)† |
| Diagnosis of mental health problems in last 12 months |  |  |  |
| No | Reference | Reference | Reference |
| Yes | 5.56 (4.20, 6.91)* | 4.84 (3.81, 5.87)* | 5.69 (4.28, 7.11)* |
| Working with COVID-19 confirmed patients or in place with high contact with COVID-19 patients |  |  |  |
| Yes | Reference | Reference | Reference |
| No | -0.86 (-2.26, 0.55) | -0.72 (-1.78, 0.34) | -0.14 (-1.62, 1.34) |
| Prefer not to say | -0.59 (-4.66, 3.47) | 0.86 (-2.22, 3.94) | 0.14 (-4.10, 4.38) |
| Changes in the number of work hours |  |  |  |
| Significantly decreased | Reference | Reference | Reference |
| Slightly decreased | -2.27 (-4.36, -0.18)* | -0.51 (-2.08, 1.06) | -1.86 (-4.02, 0.31)† |
| No change | -2.46 (-4.41, -0.50)* | 0.32 (-1.15, 1.79) | -1.53 (-3.56, 0.51) |
| Slightly increased | -1.94 (-3.96, 0.08)† | 1.03 (-0.48, 2.54) | -0.56 (-2.66, 1.54) |
| Significantly increased | -1.69 (-3.86, 0.47) | 0.81 (-0.82, 2.44) | -1.37 (-3.63, 0.89) |
| Losing job |  |  |  |
| No | Reference | Reference | Reference |
| Yes | -1.26 (-5.10, 2.58) | -1.62 (-4.41, 1.18) | -0.98 (-4.97, 3.02) |
| Loss of job of spouse/partner |  |  |  |
| No | Reference | Reference | Reference |
| Yes | -0.73 (-3.59, 2.12) | 0.45 (-1.72, 2.62) | -1.29 (-4.32, 1.73) |
| Experiencing salary cut or paycheck delay |  |  |  |
| No | Reference | Reference | Reference |
| Yes | 0.39 (-1.12, 1.91) | 0.11 (-1.04, 1.27) | -0.02 (-1.59, 1.56) |
| Experiencing financial difficulties |  |  |  |
| No | Reference | Reference | Reference |
| Yes | 1.61 (-0.07, 3.29)† | 1.12 (-0.16, 2.40)† | 0.77 (-1.00, 2.54) |
| Experiencing reduced contract renewal or other changes in job security |  |  |  |
| No | Reference | Reference | Reference |
| Yes | 1.81 (0.14, 3.47)* | 1.36 (0.10, 2.62)* | 2.59 (0.84, 4.35)* |
| Considering early retirement or being forced to retire |  |  |  |
| No | Reference | Reference | Reference |
| Yes | 4.33 (1.11, 7.56)* | 3.83 (1.32, 6.33)* | 4.75 (1.38, 8.11)* |
| Restricted access to campus, office, labs, field work, or other facilities |  |  |  |
| No | Reference | Reference | Reference |
| Yes | 1.21 (0.01, 2.41)* | 0.38 (-0.53, 1.29) | 1.07 (-0.18, 2.32)† |
| Decreased or delayed funding for research |  |  |  |
| No | Reference | Reference | Reference |
| Yes | 0.23 (-0.95, 1.41) | 0.96 (0.07, 1.85)* | 0.34 (-0.89, 1.56) |
| Delayed research work |  |  |  |
| No | Reference | Reference | Reference |
| Yes | 0.61 (-0.44, 1.66) | 0.23 (-0.57, 1.02) | 0.83 (-0.26, 1.93) |
| Challenge in recruitment of research participants |  |  |  |
| No | Reference | Reference | Reference |
| Yes | 0.80 (-0.29, 1.89) | 0.24 (-0.58, 1.07) | 0.95 (-0.19, 2.09) |
| Elimination or restructuring of department of institution |  |  |  |
| No | Reference | Reference | Reference |
| Yes | -0.09 (-1.72, 1.53) | 0.43 (-0.81, 1.67) | -0.4 (-2.09, 1.29) |
| Poor workspace or work condition at home |  |  |  |
| No | Reference | Reference | Reference |
| Yes | 2.24 (1.06, 3.42)* | 1.82 (0.93, 2.72)* | 2.84 (1.61, 4.08)* |
| Restriction on work travels |  |  |  |
| No | Reference | Reference | Reference |
| Yes | -0.99 (-2.21, 0.23) | -0.68 (-1.61, 0.24) | -0.90 (-2.16, 0.37) |
| Increased demands for childcare/eldercare |  |  |  |
| No | Reference | Reference | Reference |
| Yes | -0.69 (-1.86, 0.48) | -0.26 (-1.16, 0.63) | -0.28 (-1.51, 0.94) |
| Increased demands for domestic work |  |  |  |
| No | Reference | Reference | Reference |
| Yes | -0.01 (-1.09, 1.07) | 0.31 (-0.52, 1.13) | 0.26 (-0.88, 1.39) |

*: Significant at a significance level of 0.05. †: Significant at a significance level of 0.1. Participants with missing data were omitted.
