# Supplementary material for: Differences in self-perception of productivity and mental health among the STEMM-field scientists during the COVID-19 pandemic by sex and status as a parent: A survey in six languages
Source: PLoS One. 2022 Jul 1;17(7):e0269834. doi: 10.1371/journal.pone.0269834 (PMC9249185; doi:10.1371/journal.pone.0269834)
Supplement: S5 Table — (DOCX) [file pone.0269834.s005.docx]

**S5 Table. Results of multivariate regression analysis for DASS-21 scores of depression, anxiety, and stress for the participants in Europe (*n*=1,555).**

| Variable | Beta (95% CI) | | |
| --- | --- | --- | --- |
|  | DASS – Depression score | DASS – anxiety score | DASS – Stress score |
| Employment |  |  |  |
| Currently unemployed | Reference | Reference | Reference |
| Currently employed | 1.40 (-0.97, 3.78) | 0.76 (-0.78, 2.31) | 1.45 (-0.87, 3.76) |
| Marital status |  |  |  |
| Single | Reference | Reference | Reference |
| Divorced/widowed/separated | -1.31 (-3.04, 0.42) | -0.29 (-1.42, 0.84) | -1.87 (-3.55, -0.18)* |
| Living with a partner | -1.34 (-2.64, -0.05)* | -0.44 (-1.29, 0.4) | -0.44 (-1.68, 0.81) |
| Married | -1.32 (-2.5, -0.15)* | 0.24 (-0.52, 1.01) | -0.53 (-1.65, 0.59) |
| Early-career status |  |  |  |
| No | Reference | Reference | Reference |
| Yes | -0.13 (-1.05, 0.78) | 0.15 (-0.45, 0.75) | -0.43 (-1.32, 0.45) |
| Working in the fields involving lab experiments, bench science work, wet-science, and living organisms |  |  |  |
| No | Reference | Reference | Reference |
| Yes | 0.22 (-0.55, 1.00) | 0.56 (0.05, 1.06)* | 0.16 (-0.58, 0.91) |
| Sex |  |  |  |
| Male | Reference | Reference | Reference |
| Female | 0.09 (-0.69, 0.86) | 0.57 (0.06, 1.08)* | 1.34 (0.59, 2.10)* |
| Status as a parent of children age <18 years |  |  |  |
| No | Reference | Reference | Reference |
| Yes | -0.33 (-1.34, 0.68) | -0.30 (-0.96, 0.36) | 0.23 (-0.65, 1.11) |
| Age (years) |  |  |  |
| 19–29 | Reference | Reference | Reference |
| 30–59 | -1.07 (-2.79, 0.65) | -1.13 (-2.25, -0.01)* | 0.52 (-1.12, 2.17) |
| ≥60 | -2.81 (-4.84, -0.78)* | -2.27 (-3.58, -0.95)* | -1.71 (-3.65, 0.23)† |
| Loss of family due to COVID-19 |  |  |  |
| Yes | Reference | Reference | Reference |
| No | -0.44 (-1.24, 0.36) | -0.63 (-1.16, -0.11)* | -0.54 (-1.31, 0.24) |
| Prefer not to say | 3.58 (-1.62, 8.78) | 2.03 (-1.37, 5.43) | 0.14 (-4.82, 5.11) |
| Diagnosis of mental health problems in last 12 months |  |  |  |
| No | Reference | Reference | Reference |
| Yes | 5.80 (4.76, 6.85)* | 4.77 (4.09, 5.45)* | 5.35 (4.36, 6.35)* |
| Working with COVID-19 confirmed patients or in place with high contact with COVID-19 patients |  |  |  |
| Yes | Reference | Reference | Reference |
| No | -0.50 (-1.77, 0.77) | -1.26 (-2.10, -0.43)* | -0.96 (-2.19, 0.26) |
| Prefer not to say | 0.06 (-3.77, 3.90) | -0.11 (-2.62, 2.40) | -2.03 (-5.70, 1.64) |
| Changes in the number of work hours |  |  |  |
| Significantly decreased | Reference | Reference | Reference |
| Slightly decreased | -2.13 (-4.13, -0.12)* | -0.87 (-2.18, 0.45) | -2.5 (-4.42, -0.57)* |
| No change | -2.93 (-4.80, -1.06)* | -0.85 (-2.08, 0.38) | -3.13 (-4.91, -1.35)* |
| Slightly increased | -2.77 (-4.64, -0.90)* | -0.70 (-1.93, 0.53) | -2.76 (-4.55, -0.97)* |
| Significantly increased | -2.27 (-4.18, -0.35)* | -0.17 (-1.43, 1.09) | -0.65 (-2.49, 1.18) |
| Losing job |  |  |  |
| No | Reference | Reference | Reference |
| Yes | 3.57 (-0.01, 7.14)† | 1.66 (-0.63, 3.94) | 3.32 (-0.12, 6.76)† |
| Loss of job of spouse/partner |  |  |  |
| No | Reference | Reference | Reference |
| Yes | 1.80 (-0.54, 4.13) | 2.01 (0.48, 3.54)* | 0.64 (-1.59, 2.87) |
| Experiencing salary cut or paycheck delay |  |  |  |
| No | Reference | Reference | Reference |
| Yes | 0.20 (-1.55, 1.96) | 0.27 (-0.88, 1.41) | -0.77 (-2.47, 0.93) |
| Experiencing financial difficulties |  |  |  |
| No | Reference | Reference | Reference |
| Yes | 2.80 (1.32, 4.28)* | 1.99 (1.03, 2.95)* | 2.14 (0.73, 3.55)* |
| Experiencing reduced contract renewal or other changes in job security |  |  |  |
| No | Reference | Reference | Reference |
| Yes | 2.76 (1.39, 4.12)* | 1.10 (0.21, 1.99)* | 2.61 (1.29, 3.93)* |
| Considering early retirement or being forced to retire |  |  |  |
| No | Reference | Reference | Reference |
| Yes | -0.17 (-2.36, 2.03) | 0.27 (-1.14, 1.68) | -0.79 (-2.86, 1.29) |
| Restricted access to campus, office, labs, field work, or other facilities |  |  |  |
| No | Reference | Reference | Reference |
| Yes | 0.09 (-0.98, 1.16) | -0.60 (-1.30, 0.10)† | 0.54 (-0.50, 1.57) |
| Decreased or delayed funding for research |  |  |  |
| No | Reference | Reference | Reference |
| Yes | 0.81 (-0.05, 1.68)† | 0.73 (0.16, 1.29)* | 1.10 (0.26, 1.93)* |
| Delayed research work |  |  |  |
| No | Reference | Reference | Reference |
| Yes | 0.46 (-0.35, 1.26) | -0.43 (-0.95, 0.10) | 0.14 (-0.63, 0.92) |
| Challenge in recruitment of research participants |  |  |  |
| No | Reference | Reference | Reference |
| Yes | -0.80 (-1.62, 0.02)† | -0.32 (-0.86, 0.21) | -0.03 (-0.82, 0.76) |
| Elimination or restructuring of department of institution |  |  |  |
| No | Reference | Reference | Reference |
| Yes | 0.72 (-0.57, 2.01) | 1.01 (0.16, 1.85)* | 0.76 (-0.48, 2.01) |
| Poor workspace or work condition at home |  |  |  |
| No | Reference | Reference | Reference |
| Yes | 2.30 (1.47, 3.13)* | 0.86 (0.32, 1.41)* | 2.21 (1.42, 3.01) |
| Restriction on work travels |  |  |  |
| No | Reference | Reference | Reference |
| Yes | -0.90 (-1.83, 0.02)† | -0.61 (-1.22, -0.01)* | -0.73 (-1.64, 0.17) |
| Increased demands for childcare/eldercare |  |  |  |
| No | Reference | Reference | Reference |
| Yes | -0.52 (-1.56, 0.51) | -0.27 (-0.95, 0.40) | 0.70 (-0.30, 1.69) |
| Increased demands for domestic work |  |  |  |
| No | Reference | Reference | Reference |
| Yes | 0.20 (-0.61, 1.01) | 0.45 (-0.08, 0.98)† | 0.26 (-0.53, 1.04) |

*: Significant at a significance level of 0.05. †: Significant at a significance level of 0.1. Participants with missing data were omitted.
