# Supplementary material for: Differences in self-perception of productivity and mental health among the STEMM-field scientists during the COVID-19 pandemic by sex and status as a parent: A survey in six languages
Source: PLoS One. 2022 Jul 1;17(7):e0269834. doi: 10.1371/journal.pone.0269834 (PMC9249185; doi:10.1371/journal.pone.0269834)
Supplement: S6 Table — (DOCX) [file pone.0269834.s006.docx]

**S6 Table. Results of multivariate regression analysis for DASS-21 scores of depression, anxiety, and stress for the participants in North America (*n*=1,302).**

| Variable | Beta (95% CI) | | |
| --- | --- | --- | --- |
|  | DASS – Depression score | DASS – anxiety score | DASS – Stress score |
| Employment |  |  |  |
| Currently unemployed | Reference | Reference | Reference |
| Currently employed | -0.01 (-2.64, 2.62) | -0.65 (-2.40, 1.10) | -0.08 (-2.58, 2.42) |
| Marital status |  |  |  |
| Single | Reference | Reference | Reference |
| Divorced/widowed/separated | 0.35 (-1.68, 2.38) | -0.12 (-1.44, 1.2) | -0.15 (-2.06, 1.77) |
| Living with a partner | -0.49 (-2.4, 1.42) | 0.12 (-1.12, 1.37) | 2.23 (0.43, 4.02)* |
| Married | 0.04 (-1.34, 1.41) | -0.06 (-0.95, 0.84) | 1.08 (-0.22, 2.37) |
| Early-career status |  |  |  |
| No | Reference | Reference | Reference |
| Yes | 0.08 (-0.89, 1.06) | 0.3 (-0.33, 0.94) | 0.48 (-0.44, 1.41) |
| Working in the fields involving lab experiments, bench science work, wet-science, and living organisms |  |  |  |
| No | Reference | Reference | Reference |
| Yes | 0.53 (-0.29, 1.35) | 0.30 (-0.24, 0.84) | 0.28 (-0.50, 1.07) |
| Sex |  |  |  |
| Male | Reference | Reference | Reference |
| Female | -0.32 (-1.17, 0.52) | -0.09 (-0.64, 0.47) | 0.68 (-0.12, 1.48)† |
| Status as a parent of children age <18 years |  |  |  |
| No | Reference | Reference | Reference |
| Yes | -2.43 (-3.68, -1.17)* | -0.32 (-1.13, 0.50) | -1.72 (-2.91, -0.53)* |
| Age (years) |  |  |  |
| 19–29 | Reference | Reference | Reference |
| 30–59 | -3.70 (-6.81, -0.59)* | -1.79 (-3.82, 0.24)† | -1.94 (-4.90, 1.01) |
| ≥60 | -6.38 (-9.67, -3.09)* | -2.97 (-5.11, -0.82)* | -3.98 (-7.10, -0.86)* |
| Loss of family due to COVID-19 |  |  |  |
| Yes | Reference | Reference | Reference |
| No | -0.72 (-1.65, 0.21) | -0.80 (-1.41, -0.19)* | -0.91 (-1.79, -0.02)* |
| Prefer not to say | 1.74 (-3.25, 6.74) | -0.21 (-3.47, 3.05) | 0.79 (-3.97, 5.54) |
| Diagnosis of mental health problems in last 12 months |  |  |  |
| No | Reference | Reference | Reference |
| Yes | 4.83 (3.81, 5.84)* | 2.79 (2.13, 3.46)* | 4.57 (3.61, 5.52)* |
| Working with COVID-19 confirmed patients or in place with high contact with COVID-19 patients |  |  |  |
| Yes | Reference | Reference | Reference |
| No | -0.50 (-1.8, 0.79) | 0.44 (-0.40, 1.28) | -0.29 (-1.52, 0.94) |
| Prefer not to say | 2.54 (-2.3, 7.38) | 2.48 (-0.67, 5.64) | 1.10 (-3.51, 5.70) |
| Changes in the number of work hours |  |  |  |
| Significantly decreased | Reference | Reference | Reference |
| Slightly decreased | -0.76 (-2.56, 1.05) | -1.2 (-2.37, -0.03)* | -1.54 (-3.24, 0.17)† |
| No change | -2.31 (-4.06, -0.56)* | -1.52 (-2.66, -0.39)* | -2.47 (-4.12, -0.81)* |
| Slightly increased | -1.36 (-3.09, 0.38) | -1.04 (-2.17, 0.09)† | 0.01 (-1.64, 1.65) |
| Significantly increased | 0.13 (-1.69, 1.96) | -0.02 (-1.21, 1.16) | 1.63 (-0.09, 3.36)† |
| Losing job |  |  |  |
| No | Reference | Reference | Reference |
| Yes | 1.98 (-2.75, 6.71) | -5.12 (-8.2, -2.03)* | -1.25 (-5.75, 3.24) |
| Loss of job of spouse/partner |  |  |  |
| No | Reference | Reference | Reference |
| Yes | 0.23 (-1.68, 2.14) | 0.78 (-0.47, 2.02) | 0.00 (-1.81, 1.82) |
| Experiencing salary cut or paycheck delay |  |  |  |
| No | Reference | Reference | Reference |
| Yes | 0.53 (-0.60, 1.66) | -0.01 (-0.75, 0.73) | 0.00 (-1.07, 1.07) |
| Experiencing financial difficulties |  |  |  |
| No | Reference | Reference | Reference |
| Yes | 0.92 (-0.80, 2.64) | 1.14 (0.03, 2.25)* | -0.35 (-1.96, 1.26) |
| Experiencing reduced contract renewal or other changes in job security |  |  |  |
| No | Reference | Reference | Reference |
| Yes | 1.25 (-0.21, 2.72)† | 1.13 (0.19, 2.08)* | 1.97 (0.59, 3.35)* |
| Considering early retirement or being forced to retire |  |  |  |
| No | Reference | Reference | Reference |
| Yes | 3.15 (1.61, 4.69)* | 1.19 (0.19, 2.20)* | 1.34 (-0.13, 2.80)† |
| Restricted access to campus, office, labs, field work, or other facilities |  |  |  |
| No | Reference | Reference | Reference |
| Yes | 0.32 (-0.93, 1.58) | 0.76 (-0.06, 1.58)† | 0.39 (-0.80, 1.59) |
| Decreased or delayed funding for research |  |  |  |
| No | Reference | Reference | Reference |
| Yes | 0.04 (-0.88, 0.95) | 0.23 (-0.37, 0.83) | 0.18 (-0.69, 1.05) |
| Delayed research work |  |  |  |
| No | Reference | Reference | Reference |
| Yes | -0.04 (-0.91, 0.83) | -0.25 (-0.82, 0.31) | 0.12 (-0.71, 0.94) |
| Challenge in recruitment of research participants |  |  |  |
| No | Reference | Reference | Reference |
| Yes | -0.57 (-1.40, 0.26) | -0.41 (-0.96, 0.13) | 0.08 (-0.71, 0.87) |
| Elimination or restructuring of department of institution |  |  |  |
| No | Reference | Reference | Reference |
| Yes | 0.81 (-0.44, 2.06) | 0.51 (-0.30, 1.32) | 0.77 (-0.41, 1.95) |
| Poor workspace or work condition at home |  |  |  |
| No | Reference | Reference | Reference |
| Yes | 2.95 (2.06, 3.84)* | 0.46 (-0.12, 1.04) | 2.02 (1.18, 2.86)* |
| Restriction on work travels |  |  |  |
| No | Reference | Reference | Reference |
| Yes | -0.84 (-1.92, 0.23) | -0.91 (-1.62, -0.21)* | -0.79 (-1.81, 0.23) |
| Increased demands for childcare/eldercare |  |  |  |
| No | Reference | Reference | Reference |
| Yes | 1.20 (-0.06, 2.45)† | -0.55 (-1.37, 0.27) | 1.90 (0.71, 3.09)* |
| Increased demands for domestic work |  |  |  |
| No | Reference | Reference | Reference |
| Yes | 0.48 (-0.37, 1.34) | 0.98 (0.42, 1.53)* | 1.04 (0.23, 1.84)* |

*: Significant at a significance level of 0.05. †: Significant at a significance level of 0.1. Participants with missing data were omitted.
