# Supplementary material for: Differences in self-perception of productivity and mental health among the STEMM-field scientists during the COVID-19 pandemic by sex and status as a parent: A survey in six languages
Source: PLoS One. 2022 Jul 1;17(7):e0269834. doi: 10.1371/journal.pone.0269834 (PMC9249185; doi:10.1371/journal.pone.0269834)
Supplement: S7 Table — (DOCX) [file pone.0269834.s007.docx]

**S7 Table. Results of multivariate regression analysis for DASS-21 scores of depression, anxiety, and stress for the participants in South America and Caribbean regions (*n*=292).**

| Variable | Beta (95% CI) | | |
| --- | --- | --- | --- |
|  | DASS – Depression score | DASS – anxiety score | DASS – Stress score |
| Employment |  |  |  |
| Currently unemployed | Reference | Reference | Reference |
| Currently employed | -0.08 (-6.41, 6.25) | 0.82 (-3.57, 5.21) | -0.99 (-8.04, 6.05) |
| Marital status |  |  |  |
| Single | Reference | Reference | Reference |
| Divorced/widowed/separated | 1.68 (-1.58, 4.94) | -0.18 (-2.44, 2.08) | 0.66 (-3, 4.32) |
| Living with a partner | 2.82 (-0.38, 6.02)† | 1.69 (-0.48, 3.86) | 2.90 (-0.56, 6.36) |
| Married | 0.10 (-2.53, 2.73) | 0.85 (-0.96, 2.66) | 2.08 (-0.8, 4.97) |
| Early-career status |  |  |  |
| No | Reference | Reference | Reference |
| Yes | 1.75 (-0.40, 3.90) | -0.56 (-2.06, 0.95) | 1.78 (-0.62, 4.17) |
| Working in the fields involving lab experiments, bench science work, wet-science, and living organisms |  |  |  |
| No | Reference | Reference | Reference |
| Yes | -1.85 (-4.03, 0.34)† | 0.76 (-0.74, 2.25) | -0.93 (-3.34, 1.48) |
| Sex |  |  |  |
| Male | Reference | Reference | Reference |
| Female | 0.47 (-1.3, 2.24) | -0.12 (-1.34, 1.10) | 0.97 (-0.97, 2.91) |
| Status as a parent of children age <18 years |  |  |  |
| No | Reference | Reference | Reference |
| Yes | 0.45 (-1.79, 2.68) | -0.78 (-2.30, 0.75) | -0.10 (-2.55, 2.35) |
| Age (years) |  |  |  |
| 19–29 | Reference | Reference | Reference |
| 30–59 | -4.40 (-8.68, -0.13)* | -2.89 (-5.87, 0.09)† | -2.68 (-7.49, 2.14) |
| ≥60 | -8.06 (-13.14, -2.97)* | -4.55 (-8.07, -1.04)* | -3.61 (-9.24, 2.02) |
| Loss of family due to COVID-19 |  |  |  |
| Yes | Reference | Reference | Reference |
| No | -0.56 (-2.36, 1.24) | -1.35 (-2.57, -0.13)* | 0.09 (-1.86, 2.04) |
| Prefer not to say | -4.64 (-18.43, 9.15) | -3.03 (-12.58, 6.52) | -10.46 (-25.74, 4.82) |
| Diagnosis of mental health problems in last 12 months |  |  |  |
| No | Reference | Reference | Reference |
| Yes | 5.38 (3.49, 7.26)* | 4.12 (2.82, 5.41)* | 6.88 (4.81, 8.96)* |
| Working with COVID-19 confirmed patients or in place with high contact with COVID-19 patients |  |  |  |
| Yes | Reference | Reference | Reference |
| No | 1.51 (-1.10, 4.13) | -1.05 (-2.86, 0.76) | 1.08 (-1.82, 3.98) |
| Prefer not to say | -2.47 (-16.64, 11.7) | -3.72 (-13.56, 6.11) | -7.09 (-22.85, 8.68) |
| Changes in the number of work hours |  |  |  |
| Significantly decreased | Reference | Reference | Reference |
| Slightly decreased | -2.12 (-5.73, 1.49) | -1.32 (-3.86, 1.21) | -1.27 (-5.27, 2.73) |
| No change | -5.03 (-9.22, -0.85)* | 0.04 (-2.88, 2.96) | -2.82 (-7.54, 1.89) |
| Slightly increased | -3.25 (-6.59, 0.10)† | -1.86 (-4.2, 0.48) | -2.27 (-5.97, 1.43) |
| Significantly increased | -3.86 (-6.99, -0.73)* | -0.67 (-2.87, 1.53) | -0.30 (-3.76, 3.17) |
| Losing job |  |  |  |
| No | Reference | Reference | Reference |
| Yes | -3.85 (-9.41, 1.72) | -2.95 (-6.79, 0.88) | -3.05 (-9.95, 3.85) |
| Loss of job of spouse/partner |  |  |  |
| No | Reference | Reference | Reference |
| Yes | 2.35 (-1.73, 6.42) | 2.78 (-0.04, 5.59)† | 3.47 (-0.89, 7.84) |
| Experiencing salary cut or paycheck delay |  |  |  |
| No | Reference | Reference | Reference |
| Yes | -0.82 (-3.41, 1.77) | 2.00 (0.19, 3.82)* | -0.11 (-2.98, 2.77) |
| Experiencing financial difficulties |  |  |  |
| No | Reference | Reference | Reference |
| Yes | 2.18 (0.04, 4.32)* | 0.22 (-1.25, 1.68) | 1.52 (-0.82, 3.86) |
| Experiencing reduced contract renewal or other changes in job security |  |  |  |
| No | Reference | Reference | Reference |
| Yes | -0.42 (-3.65, 2.82) | -0.28 (-2.47, 1.91) | 0.03 (-3.57, 3.63) |
| Considering early retirement or being forced to retire |  |  |  |
| No | Reference | Reference | Reference |
| Yes | 3.39 (-0.5, 7.28)† | 1.11 (-1.59, 3.81) | -0.28 (-4.74, 4.19) |
| Restricted access to campus, office, labs, field work, or other facilities |  |  |  |
| No | Reference | Reference | Reference |
| Yes | -0.42 (-3.51, 2.67) | 0.96 (-1.19, 3.10) | -0.89 (-4.34, 2.56) |
| Decreased or delayed funding for research |  |  |  |
| No | Reference | Reference | Reference |
| Yes | 1.40 (-0.58, 3.38) | 1.30 (-0.10, 2.70)† | 1.33 (-0.89, 3.55) |
| Delayed research work |  |  |  |
| No | Reference | Reference | Reference |
| Yes | -1.73 (-3.76, 0.30)† | -1.62 (-3.02, -0.22)* | -0.30 (-2.55, 1.94) |
| Challenge in recruitment of research participants |  |  |  |
| No | Reference | Reference | Reference |
| Yes | 1.75 (-0.18, 3.69)† | 0.37 (-0.94, 1.68) | 1.38 (-0.75, 3.51) |
| Elimination or restructuring of department of institution |  |  |  |
| No | Reference | Reference | Reference |
| Yes | 3.93 (1.41, 6.45)* | 1.42 (-0.31, 3.15) | 1.16 (-1.54, 3.87) |
| Poor workspace or work condition at home |  |  |  |
| No | Reference | Reference | Reference |
| Yes | 0.04 (-1.90, 1.97) | -0.14 (-1.47, 1.20) | 1.36 (-0.77, 3.49) |
| Restriction on work travels |  |  |  |
| No | Reference | Reference | Reference |
| Yes | -0.11 (-2.32, 2.09) | 1.60 (0.06, 3.15)* | -0.64 (-3.09, 1.80) |
| Increased demands for childcare/eldercare |  |  |  |
| No | Reference | Reference | Reference |
| Yes | -1.63 (-3.70, 0.43) | -1.11 (-2.53, 0.31) | -0.78 (-3.04, 1.47) |
| Increased demands for domestic work |  |  |  |
| No | Reference | Reference | Reference |
| Yes | 0.96 (-1.20, 3.13) | 0.53 (-0.98, 2.04) | 0.72 (-1.68, 3.11) |

*: Significant at a significance level of 0.05. †: Significant at a significance level of 0.1. Participants with missing data were omitted.
