# Supplementary material for: Differences in self-perception of productivity and mental health among the STEMM-field scientists during the COVID-19 pandemic by sex and status as a parent: A survey in six languages
Source: PLoS One. 2022 Jul 1;17(7):e0269834. doi: 10.1371/journal.pone.0269834 (PMC9249185; doi:10.1371/journal.pone.0269834)
Supplement: S8 Table — (DOCX) [file pone.0269834.s008.docx]

**S8 Table. Results of multivariate regression analysis for DASS-21 scores of depression, anxiety, and stress for the participants in Oceania (*n*=173).**

| Variable | Beta (95% CI) | | |
| --- | --- | --- | --- |
|  | DASS – Depression score | DASS – anxiety score | DASS – Stress score |
| Employment |  |  |  |
| Currently unemployed | Reference | Reference | Reference |
| Currently employed | -0.89 (-8.17, 6.39) | -1.34 (-5.88, 3.21) | 2.79 (-3.80, 9.37) |
| Marital status |  |  |  |
| Single | Reference | Reference | Reference |
| Divorced/widowed/separated | -8.78 (-14.69, -2.87)* | -3.56 (-7.38, 0.26)† | -5.48 (-10.94, -0.03)† |
| Living with a partner | 0.86 (-3.39, 5.1) | 2.17 (-0.48, 4.83) | 1.63 (-2.20, 5.46) |
| Married | -3.33 (-7.09, 0.43)† | -0.95 (-3.29, 1.4) | -0.28 (-3.70, 3.13) |
| Early-career status |  |  |  |
| No | Reference | Reference | Reference |
| Yes | 1.63 (-1.58, 4.84) | 1.53 (-0.48, 3.53) | 1.37 (-1.61, 4.35) |
| Working in the fields involving lab experiments, bench science work, wet-science, and living organisms |  |  |  |
| No | Reference | Reference | Reference |
| Yes | 1.39 (-1.21, 4.00) | -1.01 (-2.64, 0.63) | 1.95 (-0.42, 4.32) |
| Sex |  |  |  |
| Male | Reference | Reference | Reference |
| Female | 0.70 (-1.87, 3.27) | 0.49 (-1.12, 2.10) | 1.31 (-1.05, 3.67) |
| Status as a parent of children age <18 years |  |  |  |
| No | Reference | Reference | Reference |
| Yes | -0.84 (-4.31, 2.63) | -0.15 (-2.37, 2.06) | 0.01 (-3.14, 3.17) |
| Age (years) |  |  |  |
| 19–29 | Reference | Reference | Reference |
| 30–59 | -3.43 (-10.10, 3.24) | -3.67 (-7.83, 0.48)† | -4.64 (-10.69, 1.41) |
| ≥60 | -4.53 (-11.87, 2.82) | -3.31 (-7.90, 1.28) | -4.00 (-10.64, 2.64) |
| Loss of family due to COVID-19 |  |  |  |
| Yes | Reference | Reference | Reference |
| No | -0.05 (-4.24, 4.14) | -1.86 (-4.47, 0.76) | -1.05 (-4.85, 2.75) |
| Prefer not to say | - | - | - |
| Diagnosis of mental health problems in last 12 months |  |  |  |
| No | Reference | Reference | Reference |
| Yes | 10.77 (7.88, 13.66)* | 4.45 (2.66, 6.23)* | 5.75 (3.17, 8.32)* |
| Working with COVID-19 confirmed patients or in place with high contact with COVID-19 patients |  |  |  |
| Yes | Reference | Reference | Reference |
| No | -4.15 (-9.35, 1.04) | 0.32 (-2.92, 3.56) | -0.76 (-5.47, 3.95) |
| Prefer not to say | 1.98 (-3.45, 7.41) | -2.65 (-6.11, 0.80) | - |
| Changes in the number of work hours |  |  |  |
| Significantly decreased | Reference | Reference | Reference |
| Slightly decreased | 0.53 (-4.07, 5.14) | -2.65 (-6.11, 0.8) | 0.41 (-4.49, 5.31) |
| No change | 1.60 (-3.20, 6.41) | -2.8 (-5.79, 0.19)† | 0.62 (-3.55, 4.78) |
| Slightly increased | 1.67 (-3.49, 6.83) | -3 (-6.11, 0.11)† | 0.93 (-3.41, 5.28) |
| Significantly increased | -7.57 (-15.72, 0.58)† | -0.43 (-3.72, 2.86) | 2.73 (-1.95, 7.40) |
| Losing job |  |  |  |
| No | Reference | Reference | Reference |
| Yes | 0.02 (-5.76, 5.80) | -5.72 (-11.4, -0.05)† | -5.45 (-12.8, 1.91) |
| Loss of job of spouse/partner |  |  |  |
| No | Reference | Reference | Reference |
| Yes | 1.22 (-2.03, 4.48) | 3.13 (-0.46, 6.73)† | 2.60 (-2.60, 7.81) |
| Experiencing salary cut or paycheck delay |  |  |  |
| No | Reference | Reference | Reference |
| Yes | 1.67 (-2.55, 5.89) | -0.25 (-2.31, 1.81) | 0.36 (-2.59, 3.31) |
| Experiencing financial difficulties |  |  |  |
| No | Reference | Reference | Reference |
| Yes | 2.42 (-0.66, 5.50) | 5.63 (3.01, 8.25)* | 0.05 (-3.77, 3.87) |
| Experiencing reduced contract renewal or other changes in job security |  |  |  |
| No | Reference | Reference | Reference |
| Yes | 3.06 (-1.72, 7.85) | -0.53 (-2.46, 1.39) | 1.63 (-1.18, 4.44) |
| Considering early retirement or being forced to retire |  |  |  |
| No | Reference | Reference | Reference |
| Yes | 1.20 (-3.91, 6.31) | 1.60 (-1.42, 4.63) | -0.96 (-5.31, 3.40) |
| Restricted access to campus, office, labs, field work, or other facilities |  |  |  |
| No | Reference | Reference | Reference |
| Yes | 0.45 (-2.26, 3.16) | -2.31 (-5.49, 0.88) | -0.72 (-5.33, 3.89) |
| Decreased or delayed funding for research |  |  |  |
| No | Reference | Reference | Reference |
| Yes | -0.66 (-3.64, 2.32) | 0.34 (-1.36, 2.05) | 0.36 (-2.08, 2.81) |
| Delayed research work |  |  |  |
| No | Reference | Reference | Reference |
| Yes | 1.63 (-0.91, 4.17) | -0.04 (-1.91, 1.82) | -1.30 (-4.01, 1.40) |
| Challenge in recruitment of research participants |  |  |  |
| No | Reference | Reference | Reference |
| Yes | -1.85 (-4.45, 0.75) | 0.91 (-0.68, 2.51) | 0.69 (-1.60, 2.99) |
| Elimination or restructuring of department of institution |  |  |  |
| No | Reference | Reference | Reference |
| Yes | 0.66 (-2.09, 3.41) | -0.96 (-2.59, 0.66) | 1.42 (-0.94, 3.77) |
| Poor workspace or work condition at home |  |  |  |
| No | Reference | Reference | Reference |
| Yes | 1.26 (-2.21, 4.73) | 0.67 (-1.04, 2.38) | 0.92 (-1.54, 3.38) |
| Restriction on work travels |  |  |  |
| No | Reference | Reference | Reference |
| Yes | 1.44 (-1.25, 4.14) | 1.09 (-1.09, 3.27) | 1.11 (-2.15, 4.36) |
| Increased demands for childcare/eldercare |  |  |  |
| No | Reference | Reference | Reference |
| Yes | -0.78 (-3.04, 1.47) | 0.93 (-1.39, 3.25) | 2.83 (-0.44, 6.10)† |
| Increased demands for domestic work |  |  |  |
| No | Reference | Reference | Reference |
| Yes | 0.72 (-1.68, 3.11) | 1.12 (-0.56, 2.81) | 1.90 (-0.51, 4.32) |

*: Significant at a significance level of 0.05. †: Significant at a significance level of 0.1. Participants with missing data were omitted.
