# Supplementary material for: Differences in self-perception of productivity and mental health among the STEMM-field scientists during the COVID-19 pandemic by sex and status as a parent: A survey in six languages
Source: PLoS One. 2022 Jul 1;17(7):e0269834. doi: 10.1371/journal.pone.0269834 (PMC9249185; doi:10.1371/journal.pone.0269834)
Supplement: Attachment — Submitted filename: 2.PLOS_response_to_Review Comments.docx [file pone.0269834.s009.docx]

**Review Comments to the Author**

**Reviewer #1:** The manuscript is a technically sound piece of scientific research and very meticulously articulated. The methodological approaches are very comprehensive. The detailed step-by-step description of data collection and analyses is very well described. An important, comprehensive study with significant findings. The authors have declared that no competing interests exist. The online survey was totally anonymous no personal information was collected and moreover, there were no direct contacts between the participants and authors. The authors have stated that due to the nature of the research participants of the study did not agree for their data to be shared publicly therefore, supporting data will not be available.

Recommended for publishing with some very few spelling corrections.

Please correct the COVID-19 spelling on page 27 (line 428)
(line 457-458) should be “To the best of our knowledge” instead of “to our best of our knowledge

**A**: Thank you for your comments. We corrected the spelling “COIVD-19” to “COVID-19” on page 21 line 371 and page 29 line 466 and line 471. The typo for “To our best of our knowledge” was changed to “To the best of our knowledge” on page 30 line 500.

**Reviewer #2**: I want to congratulate the authors for this brilliant study.
I am happy to give it a go ahead with the following suggestions:
In general comment: I would advise the authorship to consider discussing the impacts of COVID-19 pandemic in general (in other sectors, across age groups, gender identities, different contexts) as the first paragraph of the introduction. After that, they may narrow down towards academic sector and then justify why it is important to highlight the impact of COVID-19 on academic sector (In general). After that the next paragraph may introduce gender dimensions in academia and COVID-19 impact.

**A**: Thank you for your suggestion. We added new text about the impacts of COVID-19 on work environments in general and gender differences in work, poverty, and employment in the Introduction (please see page 3 lines 46-64). In page 4 lines 69-78, we added new text to introduce major changes in work routines in academia and the importance of studying challenges for scientific productivity and health in academia. We separated the original paragraph into two paragraphs and the current second paragraph (which starts on page 4 lines 79) introduces gender dimensions in academic fields.

Specific Comment: Line 42 - : (As a reader) This sounds like until March 2020, they (WHO) were sitting on it, which is a debate and should not be triggered in our vocabulary. Suggestion: "The COVID-19 pandemic that caught the world's attention in (month, year) was declared a public health emergency of international concern by the WHO in Month, Date (Ref).

**A**: Thank you for your suggestion. Please see page 3 lines 42-44. We replaced the original sentence “The COVID-19 pandemic recognized in March 2020 by the World Health Organization” to the new text that reads “The COVID-19 pandemic that received international attention in (January 2020) was declared a public health emergency of international concern by the World Health Organization (WHO) in March 2020.”
